# Pain Assessment in Hemodialysis Patients

**DOI:** 10.7759/cureus.6903

**Published:** 2020-02-06

**Authors:** Esmira Sadigova, Sultan Ozkurt, Ahmet Ugur Yalcin

**Affiliations:** 1 Internal Medicine, Faculty of Medicine, Eskisehir Osmangazi University, Eskisehir, TUR; 2 Nephrology, Faculty of Medicine, Eskisehir Osmangazi University, Eskisehir, TUR; 3 Internal Medicine: Nephrology, Faculty of Medicine, Eskisehir Osmangazi University, Eskisehir, TUR

**Keywords:** hemodialysis, pain, residual renal function, quality of life

## Abstract

Background

Pain is a common complaint among hemodialysis (HD) patients; however, most patients are not assessed for this aspect and are not sufficiently treated. In these patients, pain is reported to be associated with a range of parameters like increased depression and disrupted quality of life (QOL). Previously residual renal function (RRF) was not assessed for associations with pain. The primary aim of the study is to evaluate the pain frequency in the Turkish HD patient population. In addition, the type, origin, and severity of chronic pain, the pain treatment ratio, and the relationship between pain, QOL, and RRF were investigated during the study.

Methods

This study included 328 HD patients. Pain assessment used the McGill Pain Questionnaire and neuropathic pain assessment used the Leeds Assessment of Neuropathic Symptoms and Signs (LANSS) scale. The correlation of pain and quality of life was evaluated with the Short Form 36 (SF-36) quality of life scale.

Results

Of patients, 244 experienced pain (74.4%), and this pain had a neuropathic character in 61.8% of these patients. Patients with pain had a longer dialysis duration than those without pain (4.00 (2.00-8.00), 3.00 (2.00-4.75), p=0.01). The most common site of pain was the lower extremities. Pain was observed more often among females and with increasing age. Only 36.4% of patients used analgesics. The quality of life of patients with pain was found to be lower. The incidence of pain was higher among patients without RRF and had more neuropathic character.

Conclusions

Pain is a significant problem for the majority of HD patients and is not effectively managed. To increase the quality of life of patients, the care team should regularly question pain symptoms, and it should be treated effectively. In this context, RRF should be regularly monitored and efforts should be made to preserve it.

## Introduction

Pain is a commonly observed complaint among hemodialysis (HD) patients [1-2]. There is relatively little information about the origin, incidence, and treatment of pain. A systematic review of the prevalence of end-stage renal disease (ESRD) symptoms reported the prevalence of pain was 47% [3]. Most patients have pain severity varying from moderate to severe [4]. Three-quarters of ESRD patients suffer from insufficiently treated or untreated pain [1,4]. This problem is due to a variety of factors: caregivers are not aware of this problem and worry about the negative effects of analgesic treatment, and patients are afraid of the side effects of medication, the extra load of daily tablets, and the potential addiction risk if opioid medications are used [5]. There is increasing awareness that pain is one of the most common problems experienced by ESRD patients, and this situation is associated with increased depression and reduced quality of life (QOL) [6-7]. Disrupted QOL among HD patients was found to be associated with a higher risk of mortality and hospitalization independent of a range of demographic and comorbid factors [8].

Residual renal function (RRF) plays an important role in clearing uremic toxins, prevents excessive volume load and the related complications of left ventricle hypertrophy (LVH) and congestive heart failure (CHF), and is associated with improved metabolic parameters [9-10]. RRF is called the “heart of peritoneal dialysis”; however, very few studies have analyzed the correlation between RRF in HD patients with mortality and other important outcomes [11-13]. Additionally, it is difficult to assess RRF, and it is measured in <5% of HD patients; as a result, the research into outcomes related to this topic is limited [14]. In the literature, we did not encounter any study researching the correlation between pain and RRF.

## Materials and methods

Patient population and demographics

This prospective cross-sectional study was completed from October 2017-May 2018 and the study included 328 patients with routine hemodialysis treatment for at least three months in three different outpatient hemodialysis units. Patients were dialyzed three times a week with a synthetic membrane, each session lasting four hours with bicarbonate dialysate. Questionnaires were applied only to patients with high cognition. Patients with a cognitive disorder, under the age of 18 years, and who did not volunteer to complete the surveys were excluded from the study. The study was conducted during a four-hour HD session and at one time by a well-trained research assistant who initially explained the study to each patient and invited patients to complete a series of questionnaires about pain and QOL. Three questionnaires were used to evaluate the frequency, origin, severity, neuropathic pain, and the relationship between pain and QOL. To retain the calcium-phosphate metabolism and maintain hemoglobin levels within the target range, patients were using phosphate-binding medications, calcitriol, paricalcitol, cinacalcet, intravenous iron therapy, and an erythropoiesis-stimulating agent. Hypertensive patients were receiving angiotensin-converting enzyme inhibitor, angiotensin receptor blocker, calcium channel blocker, beta-blocker, or combined therapy.

Data collection

Demographic data for patients in the study were obtained through patient interviews at the dialysis centers and from laboratory tests within the last three months in the database.

Demographic data included history, age, sex, renal disease etiology, dialysis duration, use of analgesics, and vascular access type. Laboratory data included complete blood count (CBC), serum calcium, phosphorus, uric acid, intact parathyroid hormone (iPTH), ferritin, serum albumin, C-reactive protein (CRP), and single pool Kt/V using the Daugirdas formula [15]. RRF was defined as at least 200 ml (one glass) urine output per day. Patients were asked if they had RRF or not by the researcher who conducted the survey. Patients who expressed at least one glass of water of urine per day were evaluated as having RRF.

Measures

For an assessment of the patient’s pain, the McGill-Melzack Pain Questionnaire (MPQ) was used and the Leeds Assessment of Neuropathic Symptoms and Signs (LANSS) scale was used for the assessment of neuropathic pain. The correlation of pain with quality of life used the Short Form 36 (SF-36) Quality of Life Scale.

The McGill Pain Questionnaire

The MPQ is the most commonly used survey for the multidimensional assessment of pain [16]. It consists of four sections. The first section inquires about the localization of pain while the second section asks what the pain resembles. There are 20 word groups with two to six varying descriptive words to define pain from the sensory, perceptual, and assessment aspects. The first 10 word groups include the sensory dimension, the next five include the perceptual dimension, the sixteenth word group is about assessment, and the last four word groups include multidimensional words showing different aspects of pain. The patient marks the appropriate words for themselves in the appropriate word group. The third section questions the correlation of pain with time and additionally what increases or reduces the pain. The fourth section asks questions to determine the severity of pain. Assessment is made on an assessment scale formed of words used to define pain severity. The lowest pain score is 0 with a maximum pain score of 78. Increases in the pain score are expected to indicate the pain severity. Chronic pain is defined as pain lasting more than three months [17].

Leeds Assessment of Neuropathic Symptoms and Signs (LANSS) Scale

LANSS is the first neuropathic pain assessment scale in the literature. Application of the assessment is easy: there are five items questioning symptoms and two clinical examinations. According to the scoring: >12 points indicates the presence of neuropathic pain, while <12 points indicates no neuropathic pain [18].

Short Form 36 (SF-36) Quality of Life Scale

SF-36 is a self-report scale investigating the eight dimensions of physical function, social function, role limitations due to physical reasons, role limitations due to emotional reasons, mental health, vitality (energy), bodily pain, and general perception of health, with 36 items. Each subdimension has points varying from 0-100, with points directly proportional to quality of life [19].

Statistical analysis

Data for continuous variables are given as mean ± standard deviation or median (Q1-Q3) and categorical variables are given as frequency and percentage. The compatibility of continuous data with normal distribution was investigated using the Shapiro-Wilk test. Independent samples were compared with the Mann-Whitney test (for two groups) for variables without normal distribution. The relationship between categorical variables was examined using the chi-square test. Data were evaluated using the SPSS version 21 statistical program (IBM Corp., Armonk, NY). P<0.05 was considered significant.

## Results

The mean age of 328 patients participating in the study was 64±11 years, with 179 males (54.6%) and 149 females (45.4%). The mean HD duration of patients was 7±6 years and the mean K/tV value was 1.52±0.15. Vascular access was an arteriovenous fistula for 216 patients (66%) and a permanent tunnel catheter for 112 patients (34%). While residual urine was present in 91 patients (27.7%), 237 patients (72.3%) had no residual urine. The demographic features of patients can be seen in Tables 1-2.

**Table 1:** Demographic characteristics of participants M: male, F: female, Kt/V: Laboratory indicators of dialysis dosage, RRF: residual renal function, AVF: arteriovenous fistula, TCC: tunneled cuffed catheter

	n=328
Age (years)	64±11
Sex (M/F)	179/149
Duration of dialysis (year)	7±6
Kt/V	1.52±0.15
RRF (present/absent)	91/237
Vascular access (AVF/TCC)	216/112

**Table 2:** CKD etiology CKD: chronic kidney disease, AA: amyloid A

CKD etiology	
Hypertension	160 (48.8%)
Diabetes mellitus	81 (24.7%)
Glomerulonephritis	18 (5.5%)
Urinary tract malignancy	17 (5.2%)
AA amyloidosis	12 (3.7%)
Polycystic kidney disease	10 (3%)
Kidney stone disease	10 (3%)
Vasculitis	3 (0.9%)
Vesicoureteral reflux	1 (0.3%)
Unknown	16 (4.9%)

Of the 328 patients, 244 had pain (74.4%). Of the 244 patients with pain complaints, 181 (74.2%) stated that the pain began to develop after routine HD treatment. Of the 244 patients with pain, 151 (61.8%) had neuropathic character pain while 93 (38.1%) had nociceptive character pain (p<0.001). The mean pain severity score for patients with pain was 67.74±19.8, indicating moderate severity. Pain was present in 81.2% of female patients and 68.7% of male patients, with statistically significantly higher rates of pain among females (p=0.01).

When patients with and without pain were compared in terms of age, the mean age of patients with pain was statistically significantly higher (65.3±10.1 and 60.2±12.7, respectively, p=0.01). The serum albumin values of patients with pain were significantly lower (3.83±0.41 and3.99±0.32, respectively, p=0.001) and serum calcium values were higher (9.1±0.57 and 8.9±0.54, respectively, p=0.058) as compared to pain-free patients. However, serum albumin and calcium values were within normal reference intervals for patients. CRP, ferritin, parathormone, hemoglobin, phosphorus, and k/tV values were similar in both groups (p>0.05). Patients with pain had a longer duration of dialysis as compared to those without pain (4.00 (2.00-8.00), 3.00(2.00-4.75), p=0.01) (Table 3). No correlation was found between Kt/V and pain severity (P=0.905).

**Table 3:** Comparison of demographic and laboratory values of patients with and without pain * Mann Whitney U Test; ** Yate’s Chi-Square Test M: male, F: female, Kt/V: laboratory indicators of dialysis dosage

	Patient with pain (n=244)	Pain-free patient (n=84)	P
Hemoglobin (g/dl)	11.30 (10.62-11.90)	11.40 (10.30-12.20)	0.514*
C-reactive protein (mg/l)	10.65 (4.77-21.60)	16.35 (4.62-24.30)	0.216*
Ferritin (ng/ml)	948.00 (518.00-1461.75)	914.00 (541.25-1431.50)	0.806*
Serum albumin (g/dl)	3.90 (3.60-4.10)	4.00 (3.80-4.20)	<0.001*
Calcium (mg/dl)	9.20 (8.80-9.50)	9.00 (8.60-9.20)	0.005*
Phosphorus (mg/dl)	4.60 (3.90-5.20)	4.70 (3.70-5.67)	0.966*
Parathyroid hormone (pg/ml)	345.00 (223.50-513.75)	385.00 (227.25-756.00)	0.161*
Age (year)	66.00 (60.00-73.00)	62.00 (53.25-68.00)	0.01*
Sex (M/F)	123/121	56/28	0.01**
Duration of dialysis (year)	4.00 (2.00-8.00)	3.00 (2.00-4.75)	0.01*
Kt/V	1.57 (1.48-1.67)	1.60 (1.45-1.67)	0.738*

Of patients with pain, 193 (79.1%) had no RRF while 51 (20.9%) were identified to have RRF, and this difference was statistically significant (p<0.001; 0.291 (0.171 - 0.493)). Patients with RRF were identified to have less incidence of neuropathic (22, 14.6%) and non-neuropathic pain (29, 31.2%) as compared to those without RRF (p=0.002; 0.376 (0.200 - 0.707)). The incidence of pain was higher in patients without RRF and more neuropathic character pain was present (Table 4).

**Table 4:** Correlation between residual renal function and pain * Yate’s chi-square test RRF: residual renal function

	Pain	Pain-free	P	neuropathic pain	nonneuropathic pain	P
RRF (+)	51 (20.9%)	40 (47.6%)	<0.001	22 (14.6%)	29 (31.2%)	0.002
RRF (-)	193 (79.1%)	44 (52.4%)		129 (85.4%)	64 (68.8%)	

Of the 244 patients with pain, 77 had diabetes mellitus (31.5%). Neuropathic pain was present in 87% of patients with diabetes mellitus while 13% had nociceptive pain (p<0.001). Among nondiabetic patients, 47.4% had neuropathic pain and 52.5% had nociceptive pain (p<0.001). The presence of DM was found to significantly affect whether the pain had neuropathic characteristics (p<0.001). The prevalence of neuropathic pain in patients with DM was 6.62 times higher than the prevalence of non-neuropathic pain (OR=6.62; 95% CI 3.18 - 14.28). Additionally, the HD duration of those with neuropathic pain was found to be longer than those with non-neuropathic pain (5(2-10) and 3(2-5), p<0.001).

When the 151 patients who have neuropathic pain were analyzed per their chronic kidney disease (CKD) etiology, 67 had diabetes mellitus (44.4%), 54 had hypertension (35.8%), nine had AA amyloidosis (6%), five had polycystic kidney disease (3.3%), four had urinary tract malignancy (2.6%), three had glomerulonephritis (2%), and one had vesicoureteral reflux (0.7%). The etiology of eight patients (5.3%) out of 151 was unknown.

When the 93 patients who had nonneuropathic pain were analyzed per their CKD etiology, 60 had hypertension (64.5%), 10 had diabetes mellitus (10.8%), seven had urinary tract malignancy (7.5%), five had glomerulonephritis (5.4%), two had AA amyloidosis (2.2%), and one had polycystic kidney disease (1.1%). The etiology of eight patients (8.6%) out of 93 was unknown.

When patients are assessed in terms of pain localization, 180 patients (69.7%) had pain in the lower extremities, 47 patients (19.3%) had pain in the upper extremities, 15 patients (6.1%) had back pain, and 12 patients (4.9%) had a headache. Vascular access among patients with pain was an arteriovenous fistula (AVF) for 154 patients (63.1%) and tunneled cuffed catheter (TCC) for 90 patients (36.9%). When the correlation between vascular access type and pain localization is assessed, among 47 patients with upper extremity pain, 42 (89.4%) had AVF vascular access (p<0.001). Of the 224 patients with pain, 89 (36.4%) were using analgesics while 155 (63.6%) were not. Of 89 patients using analgesics, 15 (16.84%) were using opioid analgesics (13 tramadol, two fentanyl), 24 (26.96%) were using tricyclic antidepressants, 35 (39.32%) were using gabapentinoid antiepileptic drugs (pregabalin, gabapentin), and 15 were using paracetamol. All parameters for quality of life of patients with pain were found to be statistically significantly low as compared to those without pain (Table 5).

**Table 5:** Comparison of quality of life of patients with and without pain * Mann-Whitney U test

	Patient with pain (n=244)	Pain-free patient (n=84)	p*
Physical function	65.00 (60.00-75.00)	75.00 (70.00-80.00)	<0.001
Physical role restriction	65.00 (50.00-75.00)	75.00 (65.00-80.00)	<0.001
Emotional role restriction	75.00 (65.00-80.00)	100.00 (81.25-100.00)	<0.001
Vitality	45.00 (35.00-45.00)	45.00 (45.00-50.00)	<0.001
Bodily pain	60.00 (50.00-65.00)	75.00 (75.00-75.00)	<0.001
Social function	55.00 (55.00-65.00)	100.00 (80.00-105.00)	<0.001
Mental health	55.00 (55.00-64.00)	70.00 (65.00-90.00)	<0.001
General health perceptions	35.00 (30.00-35.00)	40.00 (35.00-45.00)	<0.001

## Discussion

In our study, the chronic pain incidence among prevalent HD patients was 74.4%, with pain developing after dialysis treatment in 74.2% of patients. Pain was present more often in females and as patient age and dialysis duration increased, the incidence of pain increased. Only 36.4% of patients with pain used analgesics. The most common pain site was the lower extremities, and patients with upper extremity pain had high rates of AVF use. Additionally, the incidence of pain was higher among patients without RRF, the pain mainly had a neuropathic character, and the quality of life of patients with pain was found to be lower.

It is estimated that 10%-20% of the general adult population suffers from chronic pain [20]. Patients undergoing dialysis treatment suffer from many physical and emotional symptoms and display high pain prevalence, and this situation causes significant disruption of QOL [1,7-8]. The prevalence of chronic pain among HD patients was 52% in the study by Tarek et al., 50% in the study by Davison et al., and 47% in the systematic review by Murtagh et al. (range 8%-82%) [1,3,21]. In our study, chronic pain prevalence was 74.4%, which is much higher than these studies. This broad difference may be due to different definitions of chronic pain, methods used for pain assessment, differences in pain perception in different populations, and broader interpretations of inclusion criteria in studies.

Pain in HD patients is associated with increased depression and reduced QOL [6-7]. Both depression and disrupted QOL are correlated with higher risks of death and hospitalization [8,22]. In our study, we found all parameters of quality of life were significantly low in patients with pain, in accordance with the literature. It is reported that pain is not sufficiently treated in HD patients while only 1/3 of patients in our study received analgesic treatment [1,4]. This situation shows the importance of those caring for HD patients questioning pain regularly as apart of medical treatment, providing effective treatment and hence increasing quality of life.

Some studies reported no effect on pain of patient age and sex among HD patients [4,21]. The study by Caravaca et al. reported that females and older patients had more incidence of pain among HD patients, similar to our results [23]. Females have a tendency to express their complaints more than males, and this situation also supports the hypothesis that females are more sensitive to pain due to mechanisms related to both peripheral and central perception systems [24]. In the general population, the elderly are more susceptible to chronic pain and it is proposed that this situation may be due to pain formation linked to involutional changes and chronic diseases in the organism, pain being due more to degenerative changes in the locomotive system, and inorganic reasons such as fear and depression [25-26].

Different from other studies showing a correlation of pain with inflammation interestingly, c-reactive protein (CRP) values were similar in the pain and pain-free groups [21,23]. We think there is a need for more studies on this topic.

In addition to the positive effects of RRF on metabolic parameters and survival in both HD and peritoneal dialysis (PD) patients, it was shown to have a positive effect on the quality of life. The presence of RRF was found to be associated with better physical function, improved vitality, kidney disease symptoms, and fewer sleep disorders [9-11,13,27]. However, the correlation of pain with RRF was not previously evaluated. In our study, 61.8% of patients with pain had neuropathic pain, and this finding complies with literature information defining 50%-90% neuropathy in HD patients [28-30]. This neuropathy was attributed to uremic neuropathy, diabetic neuropathy, or amyloidosis [28-29]. Similar to previous studies, the dialysis duration was associated with neuropathic pain; this may be explained by an increase in the incidence of uremia, diabetes, or amyloidosis over long durations of time [29-30].

In PD patients, the contribution of RRF to total clearance of moderate molecular weight (B-2 microglobulin) and protein-linked materials (P-cresol, P-cresol sulfate, and indican), was shown to be larger than small solute clearance. Though there is not much definite evidence related to the hypothesis of uremic neuropathy development, it is proposed that it may be related to the retention of neurotoxic moderate molecular weight molecules [29]. The possibility that patients with RRF have a more effective clearance of moderate molecular weight uremic toxins may explain the lower observation of pain incidence in these patients.

Strong aspects of our study include the relatively high patient numbers and being the first study to assess the correlation of RRF with pain. The study also has some limitations. The first is that patients were from a single geographic region, the second is that there was no healthy control group, and the third is that there is no information about the use of some medications, which may affect bone mineral metabolism parameters such as the use of phosphate binders (calcium carbonate or sevelamer hydrochloride), active D3 vitamins (calcitriol), and calcimimetics (cinacalcet). Finally, it would be better to use logistic regression analysis to determine factors related to chronic pain among long-term HD patients.

In conclusion, pain is a significant problem for the majority of HD patients and is not effectively managed. To increase the quality of life of dialysis patients, it is necessary that the care team, including nephrologists, regularly inquire about pain symptoms and treat them effectively. In this context, RRF should be regularly monitored and efforts should be made to preserve it. The correlation of RRF with pain should be investigated in more studies.

## Conclusions

Pain is a significant problem for the majority of HD patients and is not effectively managed. To increase the quality of life of patients, the care team should regularly question pain symptoms and the pain should be treated effectively. In this context, RRF should be regularly monitored and efforts should be made to preserve it.
